# Employers’ paradoxical views about temporary foreign migrant workers’ health: a qualitative study in rural farms in Southern Ontario

**DOI:** 10.1186/s12939-014-0065-7

**Published:** 2014-09-10

**Authors:** Miya Narushima, Ana Lourdes Sanchez

**Affiliations:** Department of Health Sciences, Brock University, 500 Glenridge Ave, St. Catharines, ON L2S3A1 Canada

**Keywords:** Agricultural workers, Migrant farmworkers, Canada, Occupational health and safety, Health promotion, Community health, Public health, Grounded theory

## Abstract

**Background:**

The province of Ontario hosts nearly a half of Canada’s temporary foreign migrant farm workers (MFWs). Despite the essential role played by MFWs in the economic prosperity of the region, a growing body of research suggests that the workers’ occupational safety and health are substandard, and often neglected by employers. This study thus explores farm owners’ perceptions about MFWs occupational safety and general health, and their attitudes towards health promotion for their employees.

**Methods:**

Using modified grounded theory approach, we collected data through in-depth individual interviews with farm owners employing MFWs in southern Ontario, Canada. Data were analyzed following three steps (open, axial, and selective coding) to identify thematic patterns and relationships. Nine employers or their representatives were interviewed.

**Results:**

Four major overarching categories were identified: employers’ dependence on MFWs; their fragmented view of occupational safety and health; their blurring of the boundaries between the work and personal lives of the MFWs on their farms; and their reluctance to implement health promotion programs. The interaction of these categories suggests the complex social processes through which employers come to hold these paradoxical attitudes towards workers’ safety and health. There is a fundamental contradiction between what employers considered public versus personal. Despite employers’ preference to separate MFWs’ workplace safety from personal health issues, due to the fact that workers live within their employers' property, workers' private life becomes public making their personal health a business-related concern. Farmers’ conflicting views, combined with a lack of support from governing bodies, hold back timely implementation of health promotion activities in the workplace.

**Conclusions:**

In order to address the needs of MFWs in a more integrated manner, an ecological view of health, which includes the social and psychological determinants of health, by employers is necessary. Employers and other stakeholders should work collaboratively to find a common ground, harnessing expertise and resources to develop more community-based approaches. Further research and continuous dialogue are needed.

## Introduction

As in many countries, foreign migrant farm workers (MFWs) are a vulnerable and underserved population in Canada despite the rapid growth of their numbers [1]. Most MFWs come to Canada through the Temporary Foreign Worker Program (TFWP) run by the federal government. In 2012, nearly 40,000 TFW positions were approved in agriculture [2], of which approximately 20,000 were in Ontario [2,3]. Most MFWs are from the Caribbean countries and Mexico, although a growing number of workers have hailed from other countries since the TFWP was expanded in 2002. The importance of the agricultural industry to the economy of Ontario has been growing. Agriculture -- including horticulture and floriculture -- is Ontario’s second-biggest industry, a $7.8 billion-a-year operation [4]. However, the number of Canadians willing to work in agriculture has declined since the 1990s, which has led to the hiring more offshore workers [5].

Although the amount of research on MFWs’ occupational safety, general health, and work and living conditions in Canada is increasing, only a small amount of research has examined these issues from the employers’ perspectives. Given employers’ strong influence on their MFWs in both work and personal spaces, it is necessary to take into account their attitudes towards health promotion activities. In this context, we conducted a qualitative study to investigate: How do employers perceive their MFWs’ occupational safety and health? What are employers’ attitudes towards creating a healthy work/life environment? What challenges do employers face promoting the safety and health of their MFWs under the current system?

### Migrant farm workers (MFWs) in Canada

MFWs come to Canada through various streams of the federal TFWP including the “Seasonal Agricultural Worker Program” (SAWP), the “Stream for Lower-Skilled Occupations” (SLSO), the “Stream of Higher-Skilled Occupations”, and the Agricultural Stream [2]. The SAWP, which was started in 1964, is operating under bilateral agreements between Canada, Caribbean countries, and Mexico. SAWP workers are recruited by the sending countries’ governments, and guaranteed agricultural work by single Canadian employers for up to eight months per year [6]. The SLSO was launched in 2002 to fulfill a labour shortage of lower-skilled jobs including agriculture, and the Agricultural Stream was further added in 2011. These new streams allow employers to directly recruit and hire people from any country, often through private brokers [2,7,8]. Compared with the SAWP, the number of workers in the SLSO and the Agricultural Streams is still small [2].

The majority of SWAP participants are married men, landless, who have low levels of education and whose migratory work is a lifeline for their families at home; female SAWP workers number less than 3%, many of whom are single mothers [9–11]. Most MFWs spend extended periods of time in Canada. Approximately 70 to 80% of SAWP workers return to the same farms for at least 7 to 8 years [10]. However, these MFWs are perpetually “temporary” and “seasonal”, because they have no legal entitlement to apply for permanent resident status in Canada [1,12].

### Occupational safety and health of MFWs in Ontario

In Canada, although the TFWP is federally regulated, workers’ labour standards fall under the jurisdiction of each province. Therefore, the situations of occupational safety and health of MFWs vary from province to province [1]. Agriculture is one of the most dangerous occupations. It is estimated that annual population fatality rates fall somewhere between 14.6 and 25.6 per 100 000 [10,13]. However, farmworkers in Ontario were excluded from the provincial Occupational Health and Safety Act (OHSA) for a long time. In 2006, in response to reports of tragedies and claims from various stakeholders (e.g., unions, legal workers), the Ontario government finally included farmworkers under the Act. Consequently, all farmworkers, including MFWs, gained various rights including the right to “refuse unsafe work”, “receive health and safety training”, “be informed about workplace hazards”, and “be included in health and safety committees”. This legislation also assigned employers various duties such as “providing information and instruction to protect their health and safety”, “maintaining equipment in good condition”, and “notifying the Ministry of Labour of workplace fatalities and injuries” [14].

A number of studies on MFWs in Canada have found specific health and safety concerns. Those include musculoskeletal problems, machine injury, pesticide and weather related ocular and skin problems, infectious diseases such as HIV, sexual transmitted infections (STIs), and reproductive and mental health problems [3,15–17]. These studies suggest that the health and safety risks of MFWs are elevated not only by workplace hazards, but also by social and psychological determinants of health that result from their low economic and precarious status. In theory, MFWs on legal temporary work permits can access public health care and employer-sponsored workplace insurance in most Canadian provinces. Nevertheless, previous research has shown that access to health care is compromised by various structural, cultural, and personal barriers. [3,9,15–17]. What are often identified as the root causes of this vulnerability of MFWs are: the “employer-specified closed work visa”, which prevents workers’ mobility within Canada; the “naming process”, which allows employers to request workers by name to return in the following year; and the “repatriation system” which allows employers to send workers home for any reason [3,9,15–17].

MFWs’ work and living conditions pose additional challenges to health care access. The SAWP participants work on an average between 50 hours to 90 hours a week during the peak of the harvest [5,10]. The long work hours allow workers to earn in eight months what it takes three to five years to earn at home [10]. MFWs often live on their employers’ farms. Given the lack of transportation in rural communities as well as language and cultural barriers, their personal life heavily depends on their employers’ availability (e.g., for grocery shopping, going to see doctors, filling-in tax forms, etc.). Many employers extend their authority through “farm rules”, such as curfews and prohibiting overnight visitors on the farm [9,18,19]. A study found that although two-thirds of SAWP employers develop good relationships with workers, the rest were problematic [18]. According to one study, weak regulations on MFWs housing and poor enforcement of public health inspection leads to unsanitary, overcrowded, and poorly ventilated living spaces [17]. Although the body of literature on MFWs specific to southern Ontario is still small, it generally suggests that workers’ health and safety conditions are substandard; that they live in social isolation, have limited access to health services, and that their overall wellbeing is largely neglected [3,9,15–17]. Several national and regional civil society groups have been established to fight for MFWs’ rights. Their aim is to raise awareness about the workers’ struggles, hoping to ultimately effect changes in legislation and/or in practice.

### Challenges for workplace health promotion in farm work

The above-mentioned inherent dangers of farm work and the vulnerability of farmworkers life are not specific to Ontario. An international trend indicates a pattern of farmworker exceptionalism when it comes to regulations and poor enforcement of existing standards [20,21]. For example, in the United States, Liebman and Augustave [21] noted that health and safety standards on family farms are problematic, especially small farms in rural areas, which are more likely to be left to their own devices [21]. The literature on the United Kingdom describes that farm owners treat health and safety legislation like a “Pandora’s box”, best to be kept closed, which in turn discourages them from taking precautions to protect themselves and their staff [22]. Even when occupational health and safety legislation exists, as Boyd & Lees (2012) described using an Australian case study, its main focus is usually injury prevention and risk management at the workplace, not health promotion *per se* [23].

In addition to farmers’ fear of the burden caused by safety regulations, the literature mentioned other factors that may be contributing to farmers’ negative attitude towards health and safety. Those included a macho culture that supports behavior leading to excessive risk and a reluctance to seek help [24]; a general perception that safety and health are employees’ responsibilities [22]; and limited financial resources [25]. Given these challenges facing agricultural workers, programs dedicated to not just injury prevention but also to health promotion are necessary. Experts agree, however, that for these programs to succeed, a community-based approach involving genuine farmworker participation is necessary [20,21]. They also recommend an ecological view of health [25], as the health of individuals is determined by their interaction with their immediate social and physical environment as well as by the broader political and socio-economic contexts in which they live and work [25]. Thus, the ecological approach to health promotion encourages the development of partnerships among the multiple stakeholders in the community [25]. One example of such efforts in Ontario is the recent initiative taken by the Occupational Health Clinics for Ontario (OHCOW). To promote partnership building, OHCOW facilitated a dialogue among multiple cross-sector stakeholders, through which health promotion on farms was identified as a priority issue. Stakeholders agreed, however, that for the success of this initiative, some difficulties needed to be overcome, namely, the lack of coordination among parties involved (service providers, workers, and cross-sector stakeholders), and the absence of farm employers in these public forums [26]. Various assumptions have been made to explain employers’ lack of involvement in their workers’ health and safety, but their voices are seldom heard to confirm or dispel such assumptions. With this in mind, we designed the present qualitative study to give farm owners an opportunity to reflect and express their points of view on the health and workplace safety of their employees.

## Methods

We conducted a qualitative research using a modified grounded theory approach to explore the views of MFWs’ employers whose voices are largely absent from existing research [27–29]. Grounded theory approach is a helpful research design to locate patterns in participants’ accounts to construct theories that are based in the data, when no appropriate guiding framework is available [28–31]. It also helps enhance our understanding of how participants’ perspectives, attitudes, and behaviour are constructed in specific personal and social contexts. Grounded theory is constructed through the interactions between participants and researchers in their perspectives and practice [28–31]. Given the sensitive nature of the topic, data collection was done through individual interviews. The study received clearance from the Research Ethics Boards of the university where the two investigators are affiliated (File no 08–287).

### Context of the study site

Although it is rare to find contextualized information about MFWs’ health and safety in a specific location, McLaughlin’s 2009 ethnographic study in a region in southern Ontario illustrates farmers’ conditions in its geographic context. The region where McLaughlin conducted her study (and the site of the present study) is a major agricultural area in Canada, ranked first in Ontario in productivity per hectare. The leading products are greenhouse (42.6%), fruit (18.9%), poultry and eggs (17.7%), nursery (5.4%), and dairy (4.6%) [15].

According to McLaughlin (2009), the trend towards large-scale agribusiness to compete on the global market combined with unpredictable natural conditions pose tremendous challenges to the farmers in the region. As a result, nearly half of region’s farmers find it hard to live solely off farming [15]. Therefore MFWs are literally the “backbone” of the growers, a remedy for their “seasonal labour shortage” (p. 260). Although the average number of MFWs per farm was 12.6, many farmers tend to give MFWs’ work and living conditions a low priority because of worries about their own survival [15].

### Recruitment

We collected data from May to June 2009 by means of face-to-face interviews with employers. We combined convenient and theoretical sampling strategies [27]. First, we asked around for those who knew farm owners or operators. We enrolled only one participant with this strategy. Next, we called and sent emails to farms listed in business directories and the yellow pages. We also placed an advertisement in the online newsletter of one of the regional farmers’ associations. These strategies helped us to recruit two more participants. The last strategy was visiting farms in person and verbally inviting available farm owners or managers; or by leaving a letter of invitation if they were busy or didn’t answer the door. For this strategy, the first author and a research assistant drove around the region and visited a total 80 farms, thereby adding an additional six participants. Although it was difficult to achieve an ideal theoretical sampling under these conditions, we still tried to include maximum variation to identify common patterns within diverse types of farms [27]. Overall, this prolonged recruitment process gave us an opportunity to observe various reactions from many farm owners or representatives – kind, diplomatic, polite, untrusting, hostile, but in general, reluctant.

### Participants

A total of nine employers (five women and four men) from nine different farms agreed to participate in the study. They were employers or their spouses, or representatives in charge of the MFWs (e.g., a vineyard manager). Participants’ ages ranged from the late 30s to mid-60s, but the majority were in their 50s. The nine farms were located in four different areas in the region. The number of MFWs hired in these farms ranged from a few to more than a hundred. Given that the average number of MFWs per farm in the region has been reported as 12.6 [15], we managed to include small, average, and large scale farms in four different categories (i.e., winery, fruit farm, nursery, flower greenhouse). A common characteristic shared by the farms was that all hired MFWs through the SAWP. Except for two relatively new farms, all had been in the SAWP for between 20 and 40 years. Five farms hired only Mexicans workers, while three hired only Caribbean workers, and one hired both.

### Data collection

A single face-to-face interview was conducted with each of the nine employers. Interviews lasted from 90 to 120 minutes and were conducted in employers’ offices, or in the barns or the field. At the beginning of the interview, a written informed consent was obtained from each participant. Semi-structured, open-ended questions were asked. Their first purpose was to gather general information about business operations (e.g., the farm size, number of employees, history of hiring MFWs, MFWs’ roles, and their practices in regards to health and safety practices). Their second purpose was to obtain the employer’s general impressions about the SWAP system and workers; their concerns in regards to workers’ health and workplace safety; and their opinions about developing health promotion programs for MFWs. Two interviewers (the first author and her research assistant) conducted each interview together. Each interview was audio recorded and transcribed immediately afterwards. In order to ensure anonymity, participants will only be identified with their code number (i.e., 1–9).

### Data analysis

To identify thematic patterns among employers’ perceptions and experiences, data analysis was conducted following the three steps of grounded theory approach [28–32]. Once the interview with the first participant was conducted and transcribed, we began an initial data analysis using line-by-line open coding and writing memos to find themes and categories, which we then compared with those identified in the transcript of the next participant. This way we were able to identify commonalities and differences between the two interviews. We kept comparing all nine transcripts successively in the same way. When new themes and categories emerged in a previous interview, they were incorporated into the questions in the following interviews for validation [28]. Upon finishing the open-coding of all interviews, we created several matrices with key sentences representing the main categories and sub-categories that emerged from all nine interviews, and compared and interpreted the meanings to develop several main categories and sub-categories (axial coding). Then, we examined the relationship among these overarching main categories and conceptualized it in a visual diagram (selective coding) [28–32]. We incorporated a few validation strategies to increase the trustworthiness of the study: researcher triangulation, by involving two investigators who worked closely throughout the research process; member checking, by sending transcripts back to the participants to check for accuracy and content modification should they request it; and negative case analysis, by refining categories and their relationships in light of disconfirming cases until most of the cases fit. A few unique cases, which we thought worth noting for the purpose of the study, were also kept and presented as a part of this paper [28].

## Results

**Figure 1:**
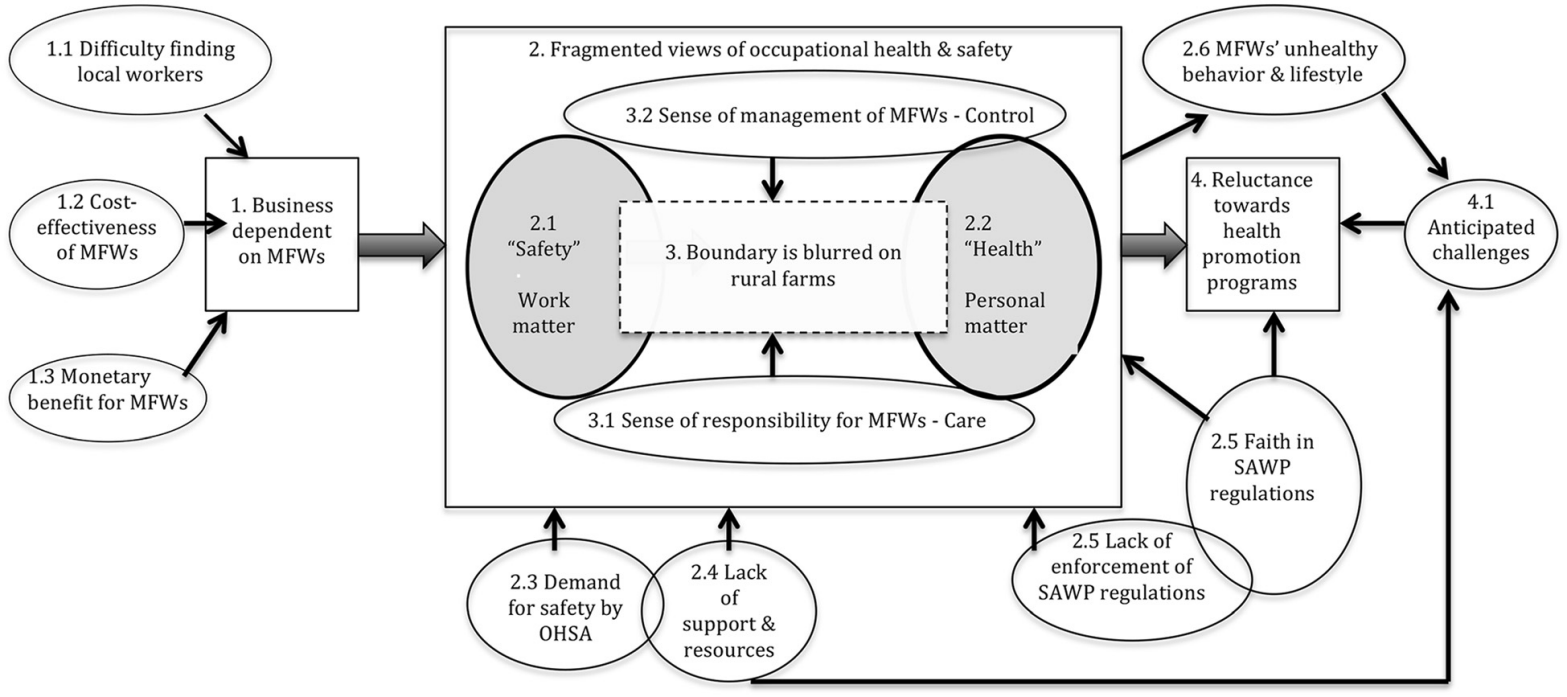
**Paradoxical views of employers about MFWs occupational safety and health.** The block arrows indicate the process through which employers form their paradoxical attitudes toward MFWs safety and health in rural farms, while the four main categories in squares indicate key contributing factors. The sub-categories shown in circles provide further contexts that lie behind each main category, which is indicated by arrows.

Although the details of participants’ experiences and views varied depending on their contexts, four main categories emerged from all nine cases. These include: 1) business dependence on MFWs, 2) fragmented views of occupational safety and health, 3) blurred boundaries between work and personal life, and 4) reluctance and perceived challenges to implement health promotion programs. A few sub-categories are nested under each of the main categories. The relationship among the main categories and the sub-categories is shown in Figure 1 (e.g., sub-category 1.1 belongs to the main-category 1).

### Business dependence on MFWs

The first category that the nine employers’ stories shared was their strong dependence on MFWs and their corresponding positive view of the SAWP system. The employers unanimously mentioned how crucial the MFWs are to operate their business: “They [MFWs] are very important. VERY, VERY important” (Participant 2); “They [MFWs] are fabulous. We could not operate without them. They are reliable, they are hard working” (Participant 7). In fact, in all farms, the MFWs, hired for six to eight months (between March and November), were the frontline laborers under a handful of Canadian supervisors. Except for one employer who had replaced half of the company’s MFWs due to workers’ health and compliance problems the previous year, 70% to 90% of the current workers in each farm were “requested returning workers” over a number of years. One employer (Participant 1) said that over a period of more than four decades, his farm had a few cases in which aged fathers were replaced by their sons.

There was a universally positive attitude towards the SWAP among employers. In the words of one participant, “it’s a win-win program for Canadian farmers and for MFWs” (Participant 2). The two main reasons cited for this positive view were: the difficulty to find Canadians, and the cost-effectiveness of hiring MFWs. The following example summarizes both:

Most of the fees are for miscellaneous items, like the airline tickets and the health insurance that the Mexican workers are paid for. But the cost to administer the program isn’t that much in the end, when you look at what it would cost to put an ad in the paper, and interview people for the same job. And the fact still remains, who would want to do what these people [MFWs] are brought up to do? Because you wouldn’t find many Canadians that would want to go out into a 100 acre vineyard with just a hand hoe, and do the work that they [MFWs] are doing (Participant 4).

Given the high unemployment ratio in the region, we asked employers if they were interested in hiring local personnel. One participant explained concerns about untrained local workers:

Of course, we do have to demonstrate that we’ve tried to hire Canadians first. I have Canadian workers as well. But, right now, anyone who wants to work is from the city, working in some factory and they were laid off; on unemployment and they want cash. Well, I’m not going to hire them. Another farm will. Only because of insurance, liability, if they’re climbing ladders and get injured … I can’t (Participant 8).

These comments illustrate the difficulties employers confront in finding seasonal, reliable, and experienced Canadian workers for farm work, and help us understand why MFWs have become an indispensable human resource. They also explain why participants used descriptions such as “predictable”, “willing to work long hours” and “very good value for money” to refer to MFWs.

When asked about the SWAP’s benefits for MFWs, employers first highlighted the financial gain. Three participants (participant 2, 4 and 9) used the exact same phrasing: “The amount offshore workers can earn during their eight months stay in Canada is equivalent to their earnings for three years in their home countries”. Other participants also emphasized that the increased income provided workers’ children better educational opportunities back home: “It is definitely helping out the third world countries”, one participant said (Participant 2). Several participants also pointed that MFWs were well protected while in Canada. One participant, however raised concerns about “casual workers” brought in by agents during peak harvest time. This employer said: “I don’t know how their situations are, they don’t look good. But we have no responsibilities for these casual workers” (Participant 8).

### Fragmented views of occupational safety and health

The second major category emerging from the employers’ stories was their tendency to view occupational safety and MFWs’ health as separate issues. As one participant clearly stated:

If it’s a work injury I get involved, if it’s a health issue of their own, then I don’t get involved, and I don’t know what’s going on, because they go to the doctor on their own, they probably don’t want me to know, or it’s personal. I don’t really want to know anyways (Participant 9).

As in this quotation, seven out of nine participants used more or less the same rhetoric: workers’ workplace safety is a business matter and therefore “our responsibility”; health is a personal matter and thus “their business”. In general, participants tended to talk more about workplace safety risks than workers’ health issues: “We really encourage them [MFWs] to be careful …especially safety, not so much their health; that’s their own concern, I think" (Participant 5). All nine participants basically stated that they were aware of the safety risks for their workers. As one participant put it:

I don’t think there are any specific hazards or aspects of health and safety that need to be highlighted, but agriculture in general is a fairly hazardous occupation, a lot of people get injured playing with agricultural machinery and things (Participant 3).

In synch with this comment, the two commonly mentioned risks were injuries and accidents related to the operation of “machines and heavy equipment” (tractors, tow-motors, etc.) and “chemicals” (e.g., pesticide). Most employers underscored that the majority of MFWs were not subject to these risks, because these operations are regulated and can only be performed by licensed workers. The employers described MFWs’ duties as “simple manual labour” – such as “hoeing”, “pruning”, “harvesting”, “packing”, “shipping” – with the associated risks being chronic physical problems such as “physical strain due to long hours in unusual postures”, “sore backs”, and “allergies”. Some participants mentioned accidents caused by “falling from ladders”, “insect bites”, “eye injury,” and “extreme weather”. Only one participant (Participant 7) said that they rotated workers so that they “didn’t have to do the same job all day long”.

Most participants stressed their adherence to the safety codes of the provincial Occupational Health and Safety Act (OHSA) introduced in 2006. The practices mentioned by most participants included “locating the government workplace safety guidebook in the office”, “providing various safety training and keeping it in the records”, and providing safety supplies and equipment such as “safety glasses”, “gloves and boots”, “winter clothes”, and “back braces”. Two employers mentioned “a health and safety committee including workers” (Participant 1 and 2). One third of participants regularly used free services from a non-profit organization providing agricultural work related safety training in multiple languages. The remaining 6 participants said that training for MFWs was done on-the-job by “certified” personnel (including the employers themselves) if necessary. It is worth noting that one third of the participants explicitly expressed their frustrations about the increasing burden on employers’ shoulders for safety training without sufficient support either from the government or farmers’ associations. One participant described their dilemma this way:

Of course, safety training is needed. But there is also a cost issue – to do it during work hours. We don’t have enough staff to implement more practical in-house kinds of training. This may be completely selfish, but I would like to see some subsidy for the training (Participant 7).

As this comment suggests, a lack of financial and human resources may prevent some employers from implementing safety training despite their understanding of how important it is and despite their dependence on their workers.

When we asked if there was any issue related to MFWs’ “health” that they thought worth mentioning in the interview, one-third of participants brought up a lack of enforcement of housing inspection by the public health department. One participant explained:

I think they [the regional public health] should be checking the employers who are not complying. I don’t think that is happening. Because I’ve heard stories from the guys [MFWs in his farm] that some of the conditions that they [MFWs] are forced to live in are really poor, and the employers are really neglectful. I think that ought to be stopped (Participant 3).

Similar to the previous comment on safety training, this comment underscores the gap between regulations and implementation. Another participant, who was the most conscientious about housing conditions for workers, shared her insights into the flaws of the regulations:

No matter how good it looks on the paper in the regulation books, it does not necessarily mean all rules are actually implemented by all employers. It is each employer’s effort and responsibility to make sure these health and safety supports are in place (Participant 5).

Although most of the participants used the regulations as safeguards for MFWs and for themselves, clearly some participants were critical about flaws in the current system.

Besides public health housing inspection, most participants said that their MFWs health was “generally pretty good” (Participant 8) or that they were “pretty physically fit” (Participant 7). This positive view was based on two reasons: Canadian government mandates a medical screening of all SAWP workers before they enter the country, and every SAWP worker has a provincial health card to access health care in Canada. Although the participants showed their confidence in the medical screening, saying “it is well taken care of by the government” (Participant 9), no participants knew what conditions and diseases the workers were screened for. A noteworthy negative case was a participant who had heard a rumor from MFWs that some returning workers from some countries can by-pass the medical screening. This participant also mentioned that, as had been reported by the media, some employers confiscate MFWs health cards and passports for “whatever reasons” (although emphasis was added: “not in this area”).

In contrast to their generally optimistic description of MFWs’ overall health, participants’ stories also revealed some concerns related to the workers’ general well-being and health, such as: “heavy drinking” (by five participants), “unprotected sex, pregnancy, and sexually-transmitted infections” (by five participants), “unhealthy diet” (by four participants), “a lack of cleaning in their houses” (by six participants), and “homesickness” (by three participants). It is important to note, however, that these concerns were categorized as workers’ “personal” and “unhealthy” behavioral and lifestyle issues. In addition, two thirds of the participants mentioned their MFWs’ tendency to not take days off despite health problems, as in the following example:

I have a worker who had a pinched nerve. So I said to him, maybe you need to just stop working for a week to let it heal. He said, “I need the money, blah, blah”. I wish you could say, “No you’re not going to work and take the week off until you feel better”. Because he wasn’t working that well anyway (Participant 5).

### Blurred boundaries between work and personal life

The third category was the tenuous, often ambiguous border between work and the personal life of MFWs in rural farms. This contrasts with the previous category – the employers’ tendency to treat health and workplace safety as separate entities. In reality, a lack of transportation, language and literacy barriers, and living on the employer’s property make it hard for both MFWs and their employers to draw a clear boundary between work and personal space. Most participants’ stories indicated that being a “boss” to the SAWP workers in rural areas put extra duties on their shoulders. A participant who was in charge of seven Mexican workers explained it this way:

It is a big responsibility. It’s a social responsibility too. Because I do have to look after them [MFWs] out of work to a degree, so if they have problems outside work, they have to have somebody who they can speak to, so they can hopefully get something done, or to point them in the right direction in terms of where they can get help (Participant 3).

Like this participant, two-thirds of the employers expressed their sense of responsibility and care for their MFWs by offering them help in their personal lives. These supports included “transportation to weekly grocery shopping”, “taking them to see doctors”, “translating medical instructions”, “helping fill in forms for health cards and tax claim”, and so forth. Some farms facilitated socializing by “sponsoring a MFWs’ soccer team”, “teaching English after work”, “having a BBQ party”, and “paying for the gas for the church van to take workers to a weekly service”. A participant explained her caring act this way: “It’s not in the contract that we have to, but that’s my job, because I’m the only one… I’m Mom for them [MFWs]” (Participant 8). Conversely, one-third of the employers responded in a rather indifferent way: “I only contract them for their labour for a certain period of time. I’m not in charge of their personal life. I am not their guardian” (Participant 9).

Regardless of the degree of appreciation for their workers, what was common among employers was the thin line dividing their sense of responsibility or care and their sense of management of MFWs’ personal lives. For example, most participants mentioned “farm rules,” including “prohibiting women visitors from staying overnight”, “monthly inspections of workers’ residences to make sure of their cleanliness”, “a curfew at 9 pm on weekdays and 11 pm on weekends”, among others. One of the reasons for “farm rules” was, according to four participants, “to respect other workers in the same house.” A participant explained it in the following terms:

As a boss, you just have to give them a code, a code to live by. Well, they can have their beer; they can have their wine; but alcoholism, no. If we suspect drugs, they go home. That’s because they cannot perform their job according to our expectations (Participant 2).

None of the participants prohibited workers drinking alcohol in their private space. However, three expressed strong concerns about workers’ occasional hangovers on work days. Several participants mentioned having to send workers back to their home countries earlier, or not calling them back the following year whenever such personal behaviour affected productivity and safety.

### Reluctance and anticipated challenges for health promotion programs

The fourth category identified was the reluctance expressed by the majority of employers towards implementing health promotion programs for MFWs in their farms. Only two participants expressed the need for more health education, particularly on nutrition, sexual health, and substance abuse. Also, the need to improve workers’ ability to communicate in English was brought up by one participant who emphasized:

I think that the language is the key. With that, you can cover a lot of things. […] When you are in a strange country, you don’t understand so many things. For me, it’s education. If you educate somebody, I believe that he will be better, he will feel more comfortable with what he is doing (Participant 1).

Most employers anticipated challenges in implementing health education programs, both on their side and the workers’, and suggested that workers were busy and focused on other things: “Workers haven’t the time or interest after working long hours” (Participant 6); “Workers were already well informed through their easy access to the media” (Participant 4). “Those guys are here to work to send money home. They don’t want to lose work hours due to training and education programs. So it should be after work” (Participant 1).

A lack of transportation came up as a big barrier for farms in remote areas: “It’s hard for them to go to these classes by bicycle unless somebody comes to pick them up” (Participant 3). Several participants mentioned cultural barriers: “If you offer these classes, it should be taught in a culturally sensitive way” (Participants 1 and 3).

As for their side, employers mentioned that the biggest obstacle was a lack of financial resources: “Who will pay for these programs?” (Participant 2, 7, and 9); “We cannot afford the hourly wage for workers if they participate in these programs during work hours” (Participant 1). Another challenge, mentioned by one participant, was the negative attitude of some growers towards workers’ sexual health education. A participant shared the following anecdote:

Knowing the number of pregnancies and STIs [sexually transmitted infections] that were around, at the annual general meeting, I had asked, with 300 people in the room, what about sexual health education? Believe it or not, they [other growers] laughed me off. They laughed me off, off of the microphone saying, ‘how uh…what we are going to teach men and women about sex?’ I just thought it was juvenile, the way the whole thing was handled, and they [other growers] didn’t get the point. They [Mexican female MFWs] don’t know the health system here. They don’t know the contacts, especially with the Spanish speaking population. Some of them are too afraid to tell anybody that they’re pregnant or they have an STI. It’s a huge oversight to me (Participant 7).

This episode depicts the challenges facing female MFWs who might need access to sexual and reproductive health education, the means for protected sex, and access to counseling or related health care.

These comments also suggest that the failure of employers in implementing health education and promotion activities in their establishments stems from various assumptions about their MFWs’ health needs as well as from challenges at their end – in particular, the lack of financial resources to compensate workers’ time if those activities occur during the work day. In addition, these comments provide a glimpse into the potential dilemma workers face: if these activities are offered during working hours but without pay, they may refuse; if offered after hours, they may be too tired or may lack the interest to attend. Moreover, due to the lack of transportation alluded to earlier, if educational activities are offered in the evenings or weekends away from the farm, even the most interested workers may find it difficult to attend. Finally, even if interested, workers need to use their free time to not just for resting and recreation but also for doing house chores and to connect with their families back home. These priorities will likely take precedence over health promotion and disease prevention activities.

Figure 1 indicates our interpretation of the process through which employers form their paradoxical attitudes toward MFWs safety and health. The basic view of the farm employers in our study is that MFWs’ occupational safety and personal health are two separate things -- a public (and business) versus a private matter. Such rhetoric strikes us as rather paradoxical given the rural farm’s unique circumstances, where MFWs have to depend on employers even for very private things, including obtaining medical care, and where SAWP employers have considerable power over their employees’ work and personal life. This paradox is problematic as well, because it leads to the employers’ reluctance or indifference to further implement health promotion programs on their farms.

## Discussion

It was difficult to recruit participants for this study. Although interpreting the reluctance to discuss these issues must be done with caution, based on the reactions we obtained from some farmers who declined participation, along with the body of literature we examined, we can infer the following two reasons. First, given the negative light often shed by the media, civil society groups, and researchers, upon farm owners’ treatment of MFWs, their unwillingness to participate may well be defensive mechanism, a natural skepticism about engaging in a conversation that could be used to generate further criticism. Second, it is possible that, as described in a British case-study [22], farmers truly fear opening the “Pandora box” of health and safety, for it could lead to overwhelming responsibilities. Or a combination of both. In any case, our findings confirm recently reported challenges in forming partnerships between farm owners and other community stakeholders [26].

Although we recruited fewer participants than we had hoped for, the stories obtained provide a rare insight into farmers’ beliefs, attitudes and difficulties. Our research shows that the process of formation of farmers’ paradoxical view about MWFs’ health and safety is compounded by four main factors. These factors are dynamically intertwined, and are also affected by the socio-economic and political context in which farmers operate.

While the MFWs’ dependence on their employers is widely known from previous studies [15,16,18], we found that employers are also heavily dependent on the MFWs for the expansion or even the survival of their business in a globally competitive market. As described by McLaughlin [15], MFWs were seen as the “backbone” of the farms by all our research participants.

Furthermore, in our study, employers reported a preference for hiring MFWs, not just because of the difficulty of finding seasonal domestic workers, but because of migrant workers’ predictability, manageability, diligence, and most importantly, cost-effectiveness. The employers also lauded the SAWP for the benefits it brings to workers and their families at home, by providing up to eight months of secure income that equals several years’ pay in their native countries [10]. We also identified nuances in the working definition of ‘local’ versus ‘foreign’ worker. Although many MFWs had been working for many years on the same farm (some farms had seen two family generations working in their fields), their status was determined “temporary”, “seasonal”, and “foreign” [1,5,33]. None of our participants seemed to question these definitions.

When asked about their workers’ health, employers affirmed their observance of the provincial Occupational Health and Safety Act (OHSA). However, and as reported by a previous study [15], our participants were generally frustrated with government demands for safety measures without providing adequate support to implement training. This suggests that tightening regulations on paper without support mechanisms and enforcement may paradoxically push safety training into the silos of individual growers rather than leading to collective solutions. This finding provides more supporting evidence for the claim that the agricultural industry has been left behind in occupational health and safety in North America [20,21]. Several employers also pointed to certain systemic flaws in regulations outlined by the SAWP. They underscored that regulation by itself does not mean protection, whereas implementation does. Some participants pointed to concrete factors contributing to unsafe workplaces; such as the lack of enforcement of housing inspection by the local public health, the bypassing of medical screening in sending countries, and some employers’ mistreatment of MFWs. These factors have also been reported in the literature [17].

The findings of our study confirmed the results of previous studies in terms of some of the barriers that MFWs face in their life on rural farms. In particular, there was resonance with topics like “the lack of transportation”, “living on the property of employers”, and “language and literacy barriers”. As previously reported, these make MFWs dependent on their employers for private matters [1,9,16,19]. Cognizant of these barriers, more than half of the employers in our study displayed a strong sense of responsibility and care for their workers, providing them with support beyond the responsibilities of the work contract. Ironically, this strong sense of responsibility sometimes turned into a paternalistic control of some aspects of workers’ private lives [12,34].

The MFWs’ occupational safety and health concerns described by employers were congruent with findings from previous studies, including “machine and pesticide related accidents and injuries”, “musculoskeletal problems”, “mental health and homesickness” and so forth [3,15,17]. In addition, we found that employers had some concerns related to MFWs’ general health including “heavy drinking”, “unprotected sex”, “unwanted pregnancy”, “unhealthy diet”, and “unsanitary houses”. Although these issues were framed by employers as “personal”, related to workers’ lifestyle and behaviour choices; they were also seen as having potential repercussions in work productivity. In a way, these findings correspond with the safety and health concerns of farmworkers in general (not just MFWs) described in the literature [20,22,25]. The employers’ fragmented views on workers’ safety and health found in this study also reflect typical employers’ (not just agricultural industry) attitudes toward workplace health and safety -- individual employees’ health as a personal issue, not a business concern [35,36].

Nevertheless, such fragmented views become paradoxical and problematic in the case of MFWs because they work and live in their employers’ rural farms. The paradox is of foreign migrant workers living under their employers’ roofs, whose occupational health is seen as a public (business) matter, but whose personal health is assumed to be a private matter. Although practical and convenient for both parties, this peculiar situation gives the employer a considerable amount of authority and scrutiny over the personal life and space of his or her tenants. For example, employers have a strong influence, intentional or otherwise, on whether or not workers can access health services, language training, religious events, recreational activities, grocery shopping, etc. Thus, personal health (broadly speaking) becomes public. Moreover, since personal health is intimately linked to job performance, in order to prevent personal health compromise or occupational health hazards, employers may implement house rules and set behavior expectations normally not imposed on independent adults. In essence, while in Canada, a MFW’s personal health is subject to business and financial (i.e. public) motivations and may therefore become an asset to be managed.

Employers are not necessarily aware of this health paradox. Neither are they familiar with the ecological view of health [25], or the notion of social and psychological determinants of health [37], as has been shown in many previous studies [3,5,9,15–17]. As described in the literature, farmers too face socio-economic and cultural struggles and barriers, and both their lives and farm operations are full of complexities and dilemmas [15,21,22,24,25]. The present study provides further evidence in this regard, yet also extends our understanding of the apparent discrepancies between farmers’ knowledge and beliefs and the actions they take that affect their workers’ health.

### Limitations and future studies

There are some limitations to this study, among which selection bias is the most relevant. Since participation rate was so low, consenting participants likely had a particular interest in having their voices heard. Also, due to the face-to-face nature of the interview, participants may have been inclined to provide socially acceptable responses. Secondly, although we could find thematic saturation because the participants were all employers of SAWP workers in one particular region, our results might have differed had we recruited more diverse participants, for example, employers from both the SAWP and SLSO streams. Future research should strive to incorporate more farmers’ voices into this complex mosaic of stories, allowing us to better understand the real and perceived disconnects between MFWs’ work and living conditions and farmers’ interests and ability to provide the best possible standards for their workers.

## Conclusions

Through this research, we have developed a coherent analysis of farmers’ attitudes and perceptions about MFWs, their health and safety. Our findings complement existing studies that have focused on MFWs’ views. It is crucial that countries accepting MFWs have in place legislation and services to ensure workers’ rights are upheld and their needs met, while improving enforcement of existing standards. MFWs are essential contributors to the prosperity of their host communities and they deserve to be supported and valued for their hard work and contributions. Regrettably, according to civil society groups and academic research, this is not always the case. One recurrent theme in past research is that MFWs’ workplace safety and health is made a low priority for the sake of profit. The voices of nine farmers willing to provide their insights reveal the complex process and challenging factors (e.g., farmers’ paradoxical views about workers’ health and wellbeing, the lack of support, resources and time constraints for safety training) which deter farmers from being more actively involved in health and safety promotion for MFWs. Taking an ecological approach to health promotion [25], it is important to develop a more supportive environment which makes it easier for farmers to participate in fostering a culture of safety and health in their farms [20,21]. As a first step, the pivotal health paradox of MFWs who live on rural farms needs to be acknowledged and addressed. At the same time, harnessing expertise and resources in the community to develop tangible support and increase political advocacy is essential to improve the wellbeing of MFWs who are economically crucial and yet structurally vulnerable [25,26]. We propose additional research along with a continued dialogue that brings together all relevant cross-sector stakeholders. This is essential if we are to find a common ground where MFWs’ health and safety can be fostered through an integrated community-based approach.

## Abbreviations

MFWs: Migrant farm workers
OHSA: Occupational health and safety act (in Ontario)
OHCOW: Occupational health clinics for Ontario workers Inc
SAWP: Seasonal agricultural worker program
SLSO: Stream for lower-skilled occupations
TFWP: The temporary foreign worker program
